# Effects and safety of vagus nerve stimulation on upper limb function in patients with stroke: a systematic review and meta-analysis

**DOI:** 10.1038/s41598-023-42077-2

**Published:** 2023-09-18

**Authors:** Auwal Abdullahi, Thomson W. L. Wong, Shamay S. M. Ng

**Affiliations:** Department of Rehabilitation Sciences, The Hong Kong Polytechnic University, Hong Kong, Special Administrative Region China

**Keywords:** Neuroscience, Neurology

## Abstract

Vagus nerve stimulation (VNS) is used to deliver electric current to stimulate the vagus nerve. The aim of this study is to carry out a systematic review and meta-analysis to determine its effects on motor function in patients with stroke. PubMED, Embase, Web of Science (WoS), and Scopus were searched. Data on time since stroke, and mean scores and standard deviation on outcomes such as level of impairment and motor function were extracted. The results showed that invasive (MD 2.66, 95% CI 1.19–4.13, P = 0.0004) and non-invasive (MD 24.16, 95% CI 23.56–24.75, P = 0.00001) VNS are superior at improving level of motor impairment than the control post intervention and at follow-up respectively. Similarly, VNS improved motor function post intervention (MD 0.28, 95% CI 0.15–0.41, P < 0.0001); and there was no significant difference in adverse events between invasive VNS and control (OR 2.15, 95% CI 0.97–4.74, P = 0.06), and between non-invasive VNS and control (OR 4.54, 95% CI 0.48–42.97, P = 0.19). VNS can be used to improve motor function in patients with stroke.

## Introduction

Stroke is a neurological disease caused by impairment in the supply of blood to the brain due to critical stenosis or occlusion and/ or rupture of the blood vessels supplying the brain^1,2^. Consequently, the survival of the cells is put in danger, and as such they may get damaged or die, and subsequently injure or lead to the death of the neighboring healthy neuronal cells^3^. Damage or death of these cells will lead to the impairment in functions of the brain, and subsequently disability in carrying out activities of daily living (ADL)^4–6^.

Currently, there are about 101 million people with stroke globally^7^. By 2050, the expected yearly incidence of stroke is 200 million^8^. Out of this number, many will survive the stroke and will eventually live with long-term disabilities especially in carrying out ADL^9–11^. In addition, although, growing evidence supports the importance of rehabilitation intervention after stroke, strategies to reduce the risk of long-term post-stroke disability beyond a year remain unclear^12^. This may be partly because of the severity of their impairment, as there are not many rehabilitation techniques that are used for severe impairment in motor function following stroke^13^. Therefore, strategies that will help enhance cortical reorganization by directly targeting the neuromodulatory systems such as the vagus nerve stimulation (VNS) are needed^14^.

Vagus nerve stimulation (VNS) is a technique used to deliver electric current to stimulate the vagus nerve^15,16^. The vagus nerve extends from the brainstem down to the colon, and in doing so, it traverses many structures that are vital to human body functions^17,18^. In addition, the nerve serves both motor and sensory functions in both the afferent and efferent regards^17,19^. The afferent function is sub-served by the afferent fibers arising from the nodose ganglion and projecting largely to the nucleus of the solitary tract (NTS)^20,21^. Projection of these afferent fibers to the NTS particularly helps to rapidly activate the cholinergic and the noradrenergic systems, which regulate various aspects of brain function, including sensory, motor and cognitive functions, and learning and memory^22–27^.

Vagus nerve stimulation (VNS) was initially started as an invasive technique where an implantable device was used to stimulate the vagus nerve; but later advances partly due to concerns about the safety of the invasive method, and the transcutaneous accessibility of the superficial branch of the vagus nerve, led to the use of a non-invasive method, where electrical current is delivered transcutaneously^14,16,28,29^. Stimulation of the vagus nerve either invasively or non-invasively, is presumed to help induce a brain environment that might increase the potential for experience-dependent plasticity^15^. However, for any rehabilitation technique to widely be accepted, what it is, for whom it is suitable, how and why it is used, and its safety need to be clearly delineated. The aim of this study is to carry out a systematic review and meta-analysis to determine the effects and adverse events of VNS; and the relationships in the reported effects and adverse events between groups in the included studies. This will help clinicians to make the most appropriate clinical decision as per as VNS is concerned. Secondly, to the best of our knowledge, this is the first study as well as a systemic review and meta-analysis to statistically determine the relationships in the reported effects and adverse events between groups in the included studies following the use of VNS in patients with stroke.

## Materials and methods

This study was registered in PROSPERO (registration number, CRD42022380312), and it was carried out using the PRISMA (Preferred Reporting Items for Systematic Reviews and Meta-Analyses) guideline.

### Eligibility criteria

Studies eligible for inclusion in this study were randomized controlled trials (RCT) that compared the use of VNS (invasive or non-invasive) with a sham VNS and/or a control intervention for the rehabilitation of upper limb function in patients with stroke. Moreover, the participants included in the studies must be 18 years old or above.

### Searching the literature

Four databases, PubMED, Embase, Web of Science (WoS), and Scopus were searched from their inceptions to December, 2022 using the key words, stroke AND vagus nerve stimulation OR auricular vagus nerve stimulation. However, the search strategies used were adapted based on the specific requirements of each database. Appendix I provides the details of the search strategy used in most of the databases. Moreover, additional search was carried out manually in the reference lists of the included studies and previous reviews on the subject matter.

One of the researchers, (AA) carried out the search independently. However, the search was verified by the two other researchers (TWLW and SSMN).

### Study selection and data extraction

Rayyan software was used to select eligible studies for inclusion^30^. The selection was performed independently by two of the researchers (AA and TWLW). These two researchers excluded some ineligible studies based on the information in their titles and abstracts. For the remaining studies, the ineligible ones were only excluded after their full texts were read by the researchers. However, for some of the studies that the two researchers could not agree on their eligibility for inclusion, the other researcher (SSMN) was consulted for discussions on how to arrive at a consensus.

In addition, data on characteristics of the study participants such as the mean age, time since stroke, type of stroke, side affected, the protocols of the VNS and the control groups including the intensity of the interventions, the outcomes assessed such as motor function, level of motor impairment and, ADL, quality of life and their mean scores post intervention and at follow-up, and the sample sizes in the studies were extracted by one of the researchers (AA). However, to ensure the data extraction was of sufficient quality, two of the other researchers (TWLW & SSMN) verified the data extracted.

The characteristics of the studies included in the review are presented in a table.

### Risks of bias and methodological quality assessment

Cochrane Risk of Bias Assessment tool and PEDro scale were used to assess the risk of bias and methodological quality of the included studies respectively. The Cochrane Risk of Bias Assessment tool is used to assess selection bias (random sequences generation and allocation concealment), performance bias (blinding of participants and personnel), detection bias (blinding of outcome assessment), attrition bias (incomplete outcome data), reporting bias (selective reporting) and any other bias not covered in the previous items of the scale^31^. The results of this assessment are presented in risks of bias graph and summary table.

The PEDro scale is an 11-items scale^32^. The first item is used to assess external validity of studies. The other ten items are used to assess internal validity of studies, and they are rated on a two-point scale, 0 and 1 which mean no and yes to a question in an item respectively. The total score from the scale is considered low or moderate or high methodological quality when it is zero to three or four to five or six to ten respectively^33–35^. The results of this assessment are presented in a table.

Both assessments were carried out independently by two of the researchers (AA and TWLW). However, where there was disagreement, the other researcher (SSMN) was consulted to help arrive at a consensus.

### Analysis of the extracted data

Narrative and quantitative syntheses were used to analyze the extracted data. The narrative synthesis involved summarizing the characteristics, risks of bias and methodological quality of the included studies. The quantitative synthesis of the effects of VNS involved the use of fixed or random effect models (where applicable) meta-analysis of the mean scores and standard deviation of the outcomes of interest, and the number of participants in the studies (for the VNS and control groups) post intervention and at follow-up.

The quantitative synthesis of the adverse events in the studies involved the use of fixed or random effect models (where applicable) meta-analysis of the number of events and sample sizes in both the VNS and the control groups post intervention and at follow-up. Similarly, the quantitative synthesis of the relationship between studies in the effects of VNS and adverse events involved meta-analysis of the correlation coefficients, r (converted from the odd ratios and mean difference of the studies) in the outcomes of interest between groups post intervention, and the sample sizes of the studies. The following formulae were used to convert odd ratios and effect size to correlation coefficient (r): $d=LogOddsRatio X \sqrt{\frac{3}{\pi }}$ and $r=\frac{d}{\sqrt{{d}^{2+a}}}$^36^. Where π = 3.14, d = effect size, r = correlation coefficient and $a=\frac{{\left(n1+n2\right)}^{2}}{n1n2}$ (where n1 and n2 = number of participants in group 1 and 2 respectively). In addition, percentage of variation across the studies due to heterogeneity (*I*^2^) was deemed significant when it is between 50 and 90% at *P* < 0.05.

Furthermore, the meta-analyses for the effects and adverse events were carried out using RevMan software; while the meta-analyses for the correlation were carried out using MedCalc^®^ software.

### Making sense of the evidence

To make sense of the evidence, body of evidence matrix of the Australian National Health and Medical Research Council's (NHMRC) evidence hierarchy was adapted^37^.

## Result

### Narrative synthesis

#### Selection of eligible studies

**Figure 1:**
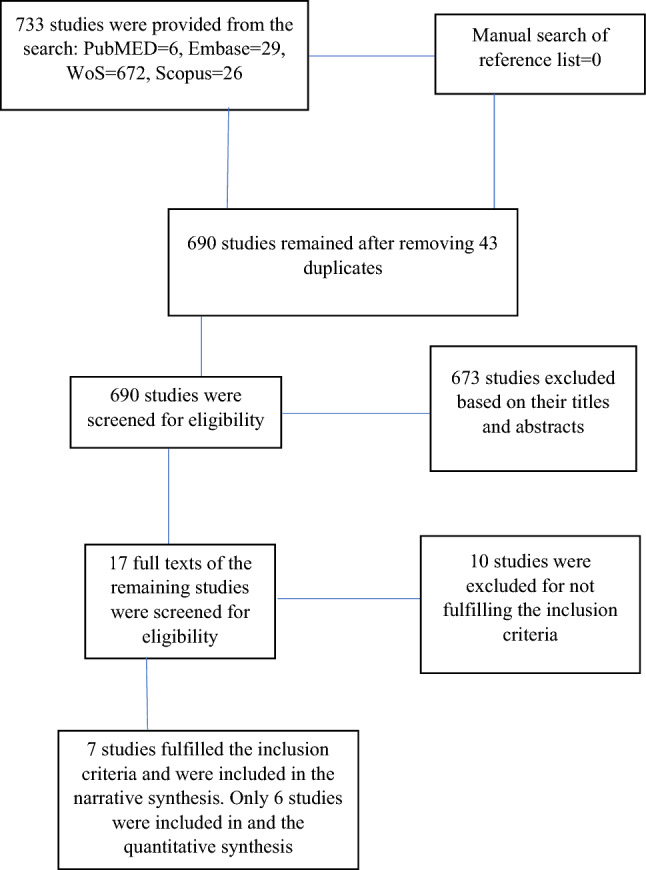
The study flowchart.

Electronic search of the databases provided a total of 733 studies. Out of this number, only seven studies were eligible for inclusion in the study^38–44^. However, in one of the studies, two papers were published^43,45^. See Fig. 1 for the details of the literature search process and the selection of the studies.

#### Characteristics of the included studies

The included studies have a total sample size of 274 patients with stroke (range 12–108), mean age range, 53.71 ± 5.88 to 69.2 ± 12.3 years and mean time since stroke range, 10.4 ± 6.9 days to about 93.71 ± 38.81 months. Out of this number, 102 were female, and the type of stroke the patients had include both ischaemic and haemorrhagic stroke. Consequently, the studies included 255 and 19 patients with ischaemic and haemorrhagic stroke respectively. Out of this, patients with ischaemic stroke were exclusively included in all the studies that used invasive VNS^39,40,43^; and one study that used non-invasive VNS^41^. However, the remaining studies that used non-invasive VNS included participants with both ischaemic and haemorrhagic stroke.

Similarly, only five studies reported the sides affected, which were 107 and 119 right and left sided hemiplegia respectively^38–44^. In addition, five studies used 125 participants with dominant hand stroke^38–40,42–44^; while, two participants in two studies were ambidextrous^40,43^.

For the period of enrolment of participants in the studies, one study each enrolled participants during the acute stage^44^; during the acute and subacute stages^41^; and during the subacute and chronic stages^40^. All the remaining studies enrolled participants during the chronic stage^38,39,42,43^.

One study included participants with moderate impairment in motor function^38^. Five studies included participants with moderate to severe impairment in motor function, a score of 15–50 on action research arm test (ARAT)^39^; and a score of 20–50 on Fugl Meyer motor assessment upper extremity (FMA-UE)^40,42–44^. However, one study did not specify the degree of impairment in motor function^41^. Similarly, the included studies used different types of VNS. Three studies used invasive VNS^39,40,43^; whereas, the remaining four studies used non-invasive VNS^38,41,42,44^.

In three studies, the stimulation parameters used were 0.8 mA, constant current, charge balanced pulses (100-μs pulse width, 30-Hz frequency^39,40,43^; in three studies, the stimulation parameters used were 600 pulses (intratrain pulse frequency = 20 Hz; pulse duration = 0*:*3 ms) ^38,41,44^; and in one study, the stimulation parameters used were single 500 ms bursts with a frequency of 30 Hz and a pulse width of 0.3 ms^42^.

In all the studies, participants in both the experimental and the control groups received upper limb rehabilitation training. In three studies, the participants received goal oriented upper limb training^39,40,43^; in two studies, the participants received conventional upper limb rehabilitation^41,43^; and in two studies, the participants received robotic rehabilitation of the upper limb^38,42^. In four studies, the stimulation was delivered simultaneously with the upper limb training^39,40,42,43^; whereas, it was delivered immediately after the training in three studies^38,41,44^.

The outcomes assessed in the studies include level of motor impairment, motor function, quantity and quality of use of the arm in the real world, hand function and/or manual dexterity, hand grip strength, muscle strength, activities of daily living (ADL), stage of recovery, spasticity, muscle electrical activity, depression, anxiety, quality of life, infarct volume and adverse events. The level of motor impairment was assessed using FMA-UE^38–44^. The motor function was assessed using Wolf motor function test (WMFT)^40–44^; and ARAT^39^. The quantity and quality of use of the arm in the real world was assessed using Motor Activity Log [MAL]^40,43^. The hand function and/or manual dexterity was assessed using Box and Block test, and 9-hole peg test^39,40^. The hand grip strength was assessed using hand-held dynamometer^39^. The muscle strength was assessed using Medical Research Council scale^42^. The activities of daily living (ADL) was assessed using Functional Independence Measure^41^. The stage of recovery was assessed using Bruunstrom recovery stage^41^. The spasticity was assessed using Modified Tardieu scale^42^. The muscle electrical activity was assessed using surface EMG^42^. The depression and anxiety were assessed using Beck Depression Index and Hospital Depression and Anxiety scale ^43,44^. The quality of life was assessed using stroke impact scale^39,40^; and stroke specific quality of life questionnaire^43^. The infarct volume was assessed using Magnetic Resonance Imaging^39^.

Both groups demonstrated improvement in most of the outcomes post intervention and at follow-up. In addition, the VNS group demonstrated a greater reduction in infarct volume post intervention. See Table 1 for the details of the characteristics of the included studies.

**Table 1:** Characteristics of the included studies.

References	N	Stroke duration	Mean age (years)	Intervention	Outcomes	Findings	Adverse events
Dawson et al.^39^	N = 20; VNS (n = 9, females = 2); control (n = 11, females = 2)	VNS = 1.8 ± 1.0 months; control = 1.7 ± 1.3 months	VNS = 57.9 ± 17.2; control = 60.7 ± 10.7	Participants in both groups received a 6-week course of 2-h therapy sessions, 3 times a week. During each session, participants performed at least 300–400 movements In addition, the VNS group received a 500-ms burst of VNS via an implanted electrode attached to the left vagus nerve in the left carotid sheath during each movement. Each simulation consisted of fifteen 0.8-mA, constant current, charge balanced pulses (100-μs pulse width, 30-Hz frequency)	Level of motor impairment (FMA-UE), motor function (ARAT), grip and pinch strength (hand-held dynamometer), quality of life (Stroke Impact Scale), manual dexterity (Box and Block test, and 9-hole peg test), safety (adverse events), feasibility (compliance with VNS) and infarct volume, CST overlap volume, fractional anisotropy ratio, and mean diffusivity Ratio (MRI)	Feasibility: All participants completed all their treatment session, and only one required removal of the implant Safety: Eight and three participants in VNS and control respectively reported adverse events; however, they were majorly not serious adverse event Efficacy: Only level of motor function attained a meaningful clinical improvement post intervention in the VNS group. However, level of motor impairment, motor function and quality of life significantly improved in VNS groups at one-year follow-up There was also greater reduction in infarct volume in VNS group post intervention	Vocal cord palsy, dysphagia, taste disturbance after the surgery (metallic taste), atrial fibrillation, reduced oxygen saturation, and chest pain
Capone et al.^38^	N = 12; VNS (n = 7, females = 3); control (n = 5, females = 2)	VNS = 93.71 ± 38.81 months; control = 46.00 ± 21.85 months	VNS = 53.71 ± 5.88; control = 55.60 ± 7.12	tVNS was delivered as trains lasting 30 s and composed by 600 pulses (intratrain pulse frequency = 20 Hz; pulse duration = 0.3 ms) repeated every 5 min for 60 min for 10 consecutive days VNS group: Electrodes were placed in the left external acoustic meatus at the inner side of the tragus Sham group: Electrodes were attached to the left ear lobe, an anatomical area that is outside the innervation of the auricular branch of the vagus nerve In addition, both groups received robotic therapy of the upper limb immediately after the stimulation	Level of motor impairment (FMA-UE), safety (blood pressure and heart rate) and tolerability (questions on unpleasant sensation and/ or discomfort)	VNS was safe. In addition, VNS significantly improved level of motor impairment better than the control	No any adverse event
Kimberly et al.^40^	N = 17; VNS (n = 8, females = 4); control (n = 9, females = 4)	VNS = 18 (1143) months, mean (range); control = 18 (6.3–53) months, mean (range)	VNS = 59.5 ± 7.4; control = 60.0 ± 13.5	VNS = In-clinic rehabilitation paired with active VNS (0.8 mA), 3 × a week for 6 weeks Control = In-clinic rehabilitation paired with sham VNS (0.0 mA), 3 × a week for 6 weeks	Level of motor impairment (FMA-UE), motor function (WMFT), quality and quantity of use of the limb in daily life (MAL), quality of life (SIS), manual dexterity (box and block test, and 9PHT), and safety (adverse events)	VNS was significantly superior only at improving motor function at 90 days follow-up compared to the control	Skin rediness in one patient in VNS group
Wu et al.^41^	N = 21; VNS (n = 10, females = 5); control (n = 11, females = 3)	VNS = 36*:*30 ± 9*:*23 days; control = 35*:*55 ± 6*:*47 days	VNS = 64*:*50 ± 9*:*97; control = 61*:*82 ± 10*:*63	VNS: The left auricular branch vagus nerve was stimulated by the modified dot-like electrodes that were fitted to the cymba conchae. The parameters were selected as follows: 600 pulses (intratrain pulse frequency = 20 Hz; pulse duration = 0*:*3 ms), lasting 30 s each time, stimulating once every 5 min. Stimulation was performed for 30 min per day for 15 consecutive days Control: Electrodes were fixed to the cymba conchae of the left ear without electrical stimulation After the stimulation, participants in both groups performed conventional rehabilitation training involving postural control, proprioception exercises, neuromuscular facilitation, gait training, and always at the upper limit of their capacity for 30 min each day	Level of motor impairment (FMA-UE), motor function (WMFT), activities of daily living (FIM), and recovery stage (upper limb Brunnstrom stage)	Level of motor impairment, motor function, and activities of daily living significantly improved better in VNS than the control post intervention and at follow-up	Skin rediness in one patient in VNS group
Chang et al.^42^	N = 36; VNS (n = 18, females = 5); control (n = 18, females = 3)	2.16 ± 0.39 years	59.02 ± 1.98 (27.9–81.1)	9 sessions of shoulder/elbow robotic therapy (3x/week for 3 weeks) paired with active taVNS or sham taVNS delivered in a single 500 ms bursts with a frequency of 30 Hz and a pulse width of 0.3 ms to the left cymba conchae during the onset of a visual cue for extension movements	Electrical activity of upper limb muscles (sEMG), level of motor impairment (FMA-UE), muscle power (MRC muscle power scale), motor function (WMFT), and spasticity (modified Tardieu Scale)	All outcomes significantly in both groups except in spasticity where VNS group improved significantly better post intervention. However, there was no significant difference in any of the outcomes at discharge or follow-up	No serious adverse events were reported
Dawson et al.^43^	N = 108; VNS (n = 53, females = 19); control (n = 55, females = 19)	VNS = 3.1 ± 2.3; control = 3.3 ± 2.6	VNS = 59.1 ± 10.2; control = 61.1 ± 9.2	Participants in both groups performed 30–50 repetitions, task-based, functional, individualised, and progressive upper limb exercises such as reach and grasp, gross movement, object flipping, simulated eating tasks, inserting objects, and opening/closing containers daily for 6 weeks In addition, VNS group received 0.8 mA (or 0.7 and 0.6 mA in two participants as described above), 100 μs, 30 Hz stimulation pulses, lasting 0.5 s, during each movement repetition. The control group received 0 mA pulses Both groups all performed 30 min therapist prescribed home exercises during the period. The VNS group was asked to put on their VNS during the home exercise	Level of motor impairment (FMA-UE), motor function (WMFT), quality and quantity of use of the limb in daily life (MAL), quality of life (SIS, SS-QOL, EQ-5D), and depression (BDI) Safety: adverse event (MedDRA, version 22)	All outcome improved higher in the VNS group post intervention and at follow-p compared to the control group	About 334 adverse events (163 VNS, 171 Control) were reported. However, majority were mild
Li et al.^44^	N = 60; VNS (n = 30, females = 15); control (n = 30, females = 16)	VNS = 10.8 ± 7.7 days; control = 10.4 ± 6.9 days	VNS = 69.2 ± 12.3; control = 68.3 ± 12.1	Participants in the VNS and control received active or sham VNS respectively delivered by an auricular transcutaneous electrical nerve stimulation apparatus using a 0.3-ms square pulses at 20 Hz for 30 s and repeated every 5 min, 20 min a day for 20 working days (5 days a week for 4 weeks) All participants received a 4-week course of 30-min rehabilitation therapy sessions, five times per week in the hospital	Motor function (WMFT), level of motor impairment (FMA-UE and FMA-LE), quality of life (SIS), anxiety and depression (HADS) and safety (adverse events)	All outcomes improved in both groups post intervention and at follow-up. However, the improvements were significantly higher in the VNS group	Two participants in VNS group reported skin redness

*VNS* vagus nerve stimulation, *FMA-UE* Fugl Meyer motor assessment- upper extremity, *ARAT* Action research arm test, *MRI* magnetic resonance imaging, *tVNS* transcutaneous vagus nerve stimulation, *WMFT* Wolf motor function test, *MAL* motor activity log, *SIS* stroke impact scale, *9HPT* Nine Hole Peg test, *FIM* Functional independence measure, *taVNS* transcutaneous auricular vagus nerve stimulation, *sEMG* surface electromyography, *MRC* Medical research council, *SS-QOL* stroke specific quality of life questionnaire, *EQ-5D* EURO-QOL five-dimension, *BDI* Beck depression index, *MedDRA* Medical Dictionary for Regulatory Activities, *FMA-LE* Fugl Meyer motor assessment-lower extremity, *HADS* Hospital anxiety and depression scale.

Furthermore, only five studies reported adverse events^39,40,43,44^. Although the adverse events are majorly mild or not serious; in one of the studies, they (Vocal cord palsy, dysphagia, taste disturbance after the surgery (metallic taste), atrial fibrillation, reduced oxygen saturation, and chest pain) seem to be serious^39^.

### Methodological quality and risks of bias

Four of the included studies have good methodological quality^38,41–43^; two have excellent methodological quality^40,44^; and one study has fair methodological quality^38^. Scores of < 4, 4–5, 6–8 and 9–10 are considered as poor, fair, good and excellent methodological quality respectively^46,47^. See Table 2 for the details of the methodological quality of the included studies.

**Table 2:** Methodological quality of the included studies.

Study	Eligibility criteria specified	Random allocation	Concealed allocation	Comparable subjects	Blind subjects	Blind therapists	Blind assessors	Adequate follow-up	Intention to treat analysis	Between group comparison	Point estimation and variability	Total score
Dawson et al.^39^	Yes	1	0	1	0	0	1	0	0	1	1	5/10
Capone et al.^38^	Yes	1	0	0	1	1	1	0	1	1	1	7/10
Kimberly et al.^40^	Yes	1	1	1	1	1	1	1	1	1	1	10/10
Wu et al.^41^	Yes	1	1	1	0	0	1	0	1	1	1	7/10
Chang et al.^42^	Yes	1	1	1	0	1	1	1	0	1	1	8/10
Dawson et al.^43^	Yes	1	0	1	1	1	1	1	0	1	1	8/10
Li et al.^44^	Yes	1	1	1	1	1	1	1	0	1	1	9/10

**Figure 2:**
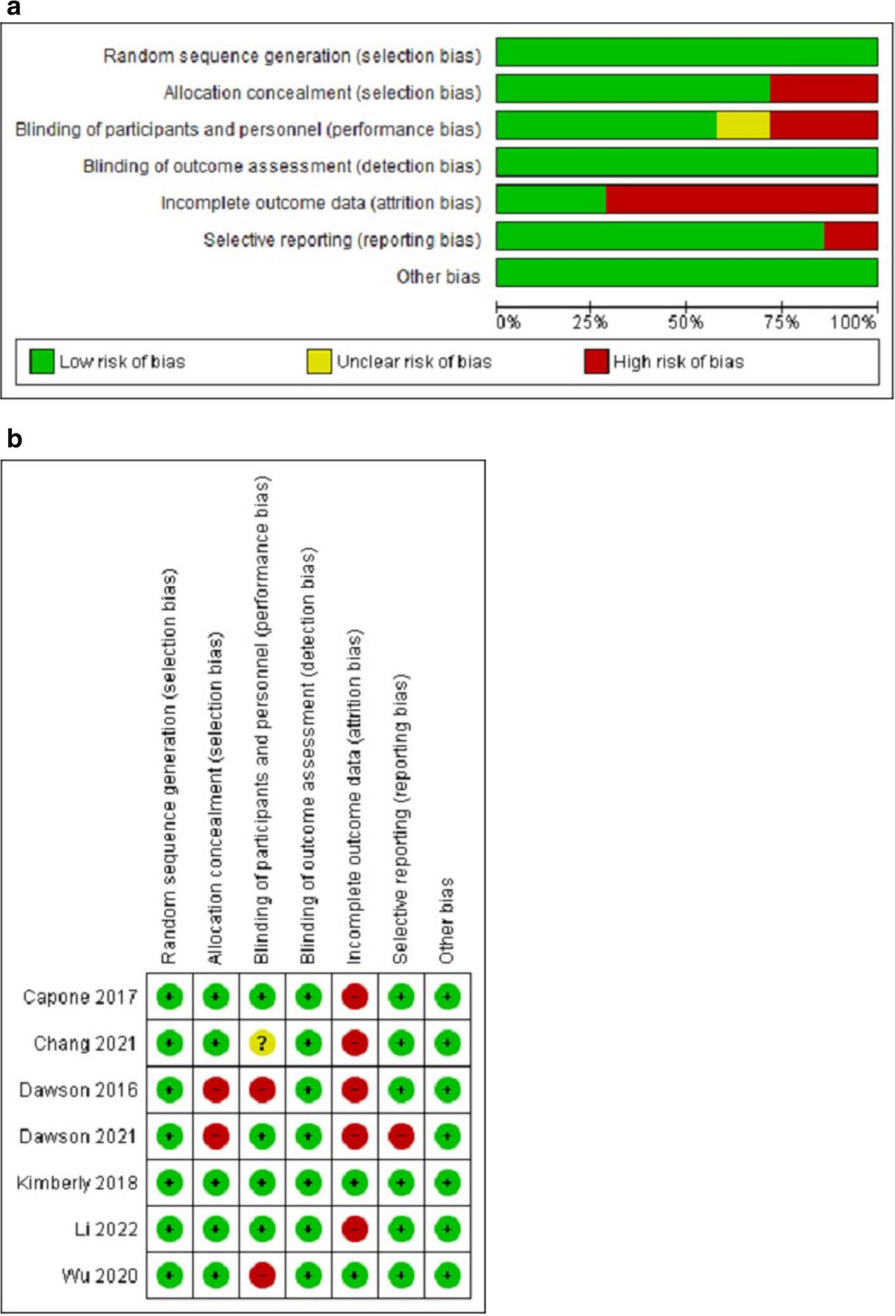
(**a**) Risks of bias graph. (**b**) Risks of bias summary.

In addition, generally, the studies have low risks of bias except in selection bias^41,42,45^; attrition bias^38,39,43,44^; and performance bias^41,42^. See Fig. 2a,b for the risks of bias graph and summary respectively.

### Quantitative synthesis

#### Effect of VNS compared with control on level of motor impairment (measured using FMA-UE)

**Figure 3:**
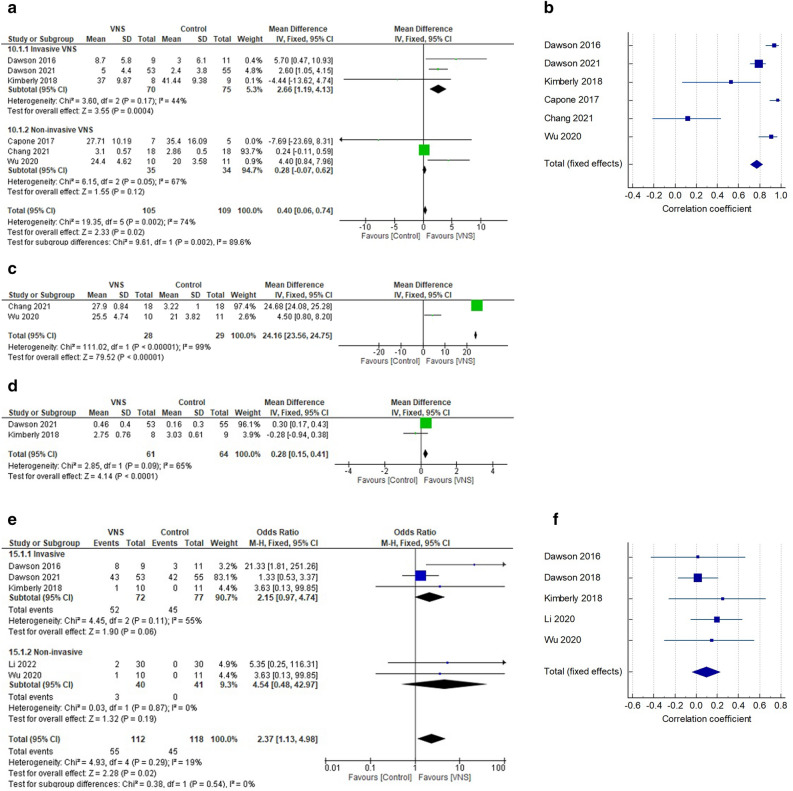
(**a**) Effect of VNS compared with control on level of motor impairment post intervention. (**b**) Relationship between VNS and control group in improving level of motor impairment post intervention. (**c**) Effect of non-invasive VNS compared with control on level of motor impairment at follow-up. (**d**) Effect of invasive VNS compared with control on motor function post intervention. (**e**) Difference in proportions of adverse events between VNS and control. (**f**) Relationship between VNS and control in adverse events.

The result showed that, invasive VNS is significantly better than control at improving level of motor impairment (MD 2.66, 95% CI 1.19–4.13, P = 0.0004) post intervention, with no significant heterogeneity between studies, (*I*^2^ = 44%, P = 0.17) (see Fig. 3a for the details of the result). However, there was a significant correlation between improvements in both groups post intervention (r = 0.77, 95% CI 0.708–0.822, P < 0.001), possibly suggesting that, upper limb rehabilitation training used in both groups contributed to the improvement immensely (see Fig. 3b for the details of the result). In addition, the relatively large confidence interval in the result of the effect, suggests uncertainty concerning the effect of invasive VNS.

For the non-invasive VNS, the result showed that, there was no significant difference between groups post intervention (MD 0.28, 95% CI − 0.07 to 0.62, P = 0.12), with significant heterogeneity between studies, *I*^2^ = 67%, P = 0.05) (see Fig. 3a for the details of the result). However, at follow-up, the result, showed that, non-invasive VNS is significantly better than control at improving level of motor impairment (MD 24.16, 95% CI 23.56–24.75, P = 0.00001), with significant heterogeneity between studies, (*I*^2^ = 99%, P < 0.00001) (see Fig. 3c for the details of the result).

#### Effect of VNS compared with control on motor function

The result showed that, invasive VNS is significantly better than control at improving motor function (MD 0.28, 95% CI 0.15–0.41, P < 0.0001) post intervention, with significant heterogeneity between studies, (*I*^*2*^ = 65%, P = 0.09) (see Fig. 3d for the details of the result).

#### Presence of adverse events

The results showed that, there was no significant difference in the presence of adverse events between invasive VNS and control (OR 2.15, 95% CI 0.97–4.74, P = 0.06), with significant heterogeneity between studies (*I*^2^ = 55%, P = 0.11); and no-invasive VNS and control (OR 4.54, 95% CI 0.48–42.97, P = 0.19) with no significant heterogeneity between studies (*I*^*2*^ = 0%, P = 0.87) (see Fig. 3e for more details). However, there was no significant correlation between groups in adverse events (r = 0.0942, 95% CI − 0.0405 to 0.225, P = 0.170), suggesting that, VNS may produce adverse events higher than the control (see Fig. 3f for more details).

### Interpretation of the evidence

The evidence seems to be excellent, satisfactorily consistent and applicable, excellently generalizable and has good clinical impact. Therefore, the body of evidence can be trusted to guide practice in most cases. See Table 3 for the body of the evidence matrix.

**Table 3:** Body of evidence matrix.

Component	Grade	Comments
1. Evidence	A-Excellent Several Level II studies	Quantity: a total of 7 studies Participants: 274 patients with stroke Level II studies: 7
2. Consistency	C-Satisfactory	There is significant heterogeneity between studies for one of the outcomes, *I*^*2*^ > 50%
3. Clinical impact	B-Good	Several studies reported that the effect attained clinically meaningful^39,40,42,43^
4. Generalizability	A-Excellent	The studied population is the same as the target population (patients with stroke)
5. Applicability	C-Satisfactory	The evidence may be applicable globally since the studies were carried out in 4 different countries (China, Italy, UK and USA) in three different continents
Recommendation	B = Body of evidence can be trusted to guide practice in most cases	

## Discussion

The aim of this study was to carry out a systematic review and meta-analysis to determine the effects, adverse events of VNS and the relationships in the reported effects and adverse events between groups in the included studies. The results showed that VNS improves outcomes such as level of motor impairment and motor function. In addition, there was no significant difference between groups in terms of adverse events. These findings are important since search for effective rehabilitation techniques for the rehabilitation of upper limb function in patients with stroke remain an important goal for clinicians, patients and their families^14^. Therefore, VNS can serve as a means to reach centrally located neurological structures to help patients with stroke recover upper limb function^17^. This is because the vagus nerve serves both motor and sensory functions, which are essential for recovery of upper limb functional activities^17,19^.

The afferent function of the vagus nerve is promoted by the afferent fibers that arise from the nodose ganglion and project majorly to the NTS^20,21^. This helps to activate the cholinergic and noradrenergic systems that are required for various normal functions of the brain^22–24,28^. This is made possible because most cholinergic and adrenergic neurons are located in subcortical regions and have axons that innervate many brain regions, including cortices and the hippocampus^48,49^. Consequently, the engagement of these neuromodulatory systems by VNS led to the prediction that brief bursts of VNS paired with sensory or motor experience could enhance cortical plasticity that was specific to the paired experience^14^.

However, from the results of the present study, there was significant correlation in improvements in level of motor impairment between the VNS and control groups, suggesting that upper limb rehabilitation training that was used in both groups may have a crucial effect on the improvement. This is because, the types of interventions used for the upper limb trainings in the included studies are known to also improve upper limb function^50,51^. Thus, VNS may be used as an adjunct therapy to other rehabilitation techniques, which re-echoes previous claim that neuromodulation techniques should be used in combination with other rehabilitation techniques^13^. Similarly, hybrid therapy, where two or more techniques are combined has been advocated during stroke rehabilitation for optimal gain^52^. In addition, there was no significant correlation between groups in presence of adverse events, suggesting that, one of the interventions may produce higher or more serious adverse events.

Another issue concerning the results of the present study that needs discussing, is the characteristics of patients with stroke who are most suitable for VNS. This is because, from the results of the included studies, the participants used were those with mild to moderate impairment in motor and cognitive functions. However, VNS is a passive technique and it does not require active performance by the patients. Similarly, it has also been reported to help improve cognitive function^53,54^. Therefore, VNS can be used for patients with stroke who have severe impairment in motor and cognitive function. This is a scientific breakthrough as there is as yet not many rehabilitation techniques used for patients with severe impairment in motor function^13^.

Another issue is that, considering the cost and potential risks of adverse events with the use of invasive VNS, especially the risk of introducing infection and having wound due to surgery, the non-invasive technique should be given a special attention especially for research. However, the non-invasive technique is still not widely used as there is no quality evidence of its superiority over control interventions^55^. Similarly, future studies should try and compare the use of invasive VNS versus non-invasive VNS for the rehabilitation upper limb function following stroke.

## Conclusion

The evidence for the use of VNS for the rehabilitation of upper limb function in patients with stroke seems to be excellent, satisfactorily consistent and applicable, excellently generalizable and has good clinical impact. Therefore, the body of evidence can be trusted to guide practice in most cases. However, further studies are needed specially to compare the effects of invasive VNS with non-invasive VNS, and the presence of adverse events following them.

### Supplementary Information

Supplementary Information.

## Data Availability

All data generated or analyzed during this study are included in this published article.
