# Supplementary material for: Effects and safety of vagus nerve stimulation on upper limb function in patients with stroke: a systematic review and meta-analysis
Source: Sci Rep. 2023 Sep 18;13:15415. doi: 10.1038/s41598-023-42077-2 (PMC10507009; doi:10.1038/s41598-023-42077-2)
Supplement: Supplementary file 1 — Supplementary Information. [file 41598_2023_42077_MOESM1_ESM.pdf]

Appendix 1: Search strategy

((((Stroke) AND (Vagus nerve stimulation)) OR (Transcutaneous vagus nerve stimulation)) OR (Auricular vagus nerve stimulation)) AND (Upper extremity)
